# The Mediating Effect of Parental Involvement on School Climate and Behavior Problems: School Personnel Perceptions

**DOI:** 10.3390/bs10080129

**Published:** 2020-08-09

**Authors:** Sónia Maria Martins Caridade, Hélder Fernando Pedrosa e Sousa, Maria Alzira Pimenta Dinis

**Affiliations:** 1 Faculty of Human and Social Sciences, University Fernando Pessoa (UFP), Praça 9 de Abril, 349, 4249-004 Porto, Portugal; 2 Permanent Observatory Violence and Crime (OPVC), University Fernando Pessoa (UFP), Praça 9 de Abril 349, 4249-004 Porto, Portugal; madinis@ufp.edu.pt; 3 Interdisciplinary Center for Gender Studies (CIEG), Higher Institute of Social and Political Sciences, University of Lisbon (ISCSP-UL), 1300-663 Lisboa, Portugal; 4 Department of Mathematics (DM.UTAD), University of Trás-os-Montes and Alto Douro (UTAD), Quinta de Prados, 5001-801 Vila Real, Portugal; hfps@utad.pt; 5 UFP Energy, Environment and Health Research Unit (FP-ENAS), University Fernando Pessoa (UFP), Praça 9 de Abril 349, 4249-004 Porto, Portugal

**Keywords:** school climate (SC), parental involvement (PI), behavior problems (BP), school personnel, mediator variables

## Abstract

As a reflection of the culture and norms of the school community, the school climate (SC) is a potential factor connected to students’ major behavior problems (BP). Parental involvement (PI) is considered as an essential factor for SC, contributing to promote good students’ educational results, as well as better social functioning. The present study aimed to analyze the mediating effect of PI on the relationship between SC and BP, taking into consideration the school personnel perceptions. A total of 329 school personnel (teachers versus no teachers) aged between 29 and 66 (*M* = 50.78, *SD* = 7.56), mainly female (79%), were integrated in the sample. Based on the perception of the school personnel, the results indicate moderate level of PI and SC, as well as the existence of different BP in the school context. The mediating effect of PI in the relationship between the SC and BP has been demonstrated. These results suggest that, if the SC and PI are improved, it could be an effective strategy to enhance the social functioning of students in the school context. This study thus contributes to a comprehensive empirical analysis of how PI can improve the relationship between the SC and the BP of Portuguese students.

## 1. Introduction

Different definitions about school climate (SC) concept (e.g., [1]) can be found in literature. For the purpose of this study, SC was defined as a wide term involving the quality and character of school life in terms of norms and values, interpersonal relationships, social interactions, and organizational processes, structures, and culture [2]. Considered a multifaceted concept, SC includes observable characteristics of schools, organizational behavior of school personnel, and shared values among students and school personnel [3]. In this sense, SC has been conceptualized as a critical factor in school life because it establishes socially acceptable behavior at school, which is able to influence and shape interactions between all school members (i.e., students, teachers, and parents) as well as their development at various levels [4].

Many different SC models have been proposed (e.g., [5,6,7]). SC has been operationalized into four main dimensions, with different elements: (i) safety, that could involve physical and emotional safety, rules and norms [7]; (ii) teaching and learning encompassing, for example, support for learning, quality instructional, social, emotional, and ethical learning (e.g., [5]); (iii) relationships, which involve respect for the community, school diversity school and collaboration, morale and connectedness (e.g., [5]); (iv) environmental–structural, which includes physical characteristics, aesthetics and space size, and resources [5], school connectedness/engagement, and physical layout and surroundings [6,7]. In the present study, only environmental–structural (e.g., access, adequate space and materials; adequate aesthetic quality and size of school; extracurricular offerings) and (ii) and relationships (e.g., mutual support and ongoing communication; school–community involvement) dimensions are considered. These two selected SC dimensions to this study are supported in the context of the literature framework. In fact, many of the SC-centered studies only address two of the above-mentioned dimensions (e.g., [5,8]) or even a single construct [9]. In addition, the literature proved the existence of a relationship between the institutional environment, interpersonal relationships, and BP, which has not been observed in other SC dimensions, that is, safety, teaching, and learning, since they were not equally considered in the analysis of the various studies [10]. The multidimensionality of the SC is very important, as it will allow one to better assess the changes to be made in the school context [11], as well as to identify the main BP disturbing the good school functioning, able to interfere with the students’ performance [12], all decisive factors to prevent the development of a delinquent trajectory [13].

For the purpose of this study, the BP concept has a broader meaning, which includes a combination of disruptive, antisocial, delinquent, deviant, and risk or externalizing behaviors [10,14], that could have serious negative consequences for adolescents or general society. Absenteeism and incivilities practiced in the school context are also considered [12]. School-centered research has recognized the existence of several BP, such as violence and indiscipline, which can be easily identified in the school context, capable of contributing to the breakdown of social attachment through the use of force and aggression [12,15], drug abuse [16,17], delinquent, antisocial behaviors, and incivilities occurring in the school or in the space surrounding schools [18,19] or even absenteeism [15].

While members of the school community, parents play an important role in the learning environment, in defining school policies and practices, as well as in the academic and social performance of students [20]. Considered as a multidimensional concept [21], PI comprises several behavioral practices of parents (e.g., parents’ style of life, parental expectations and aspirations, domestic rules and parental supervision, helping with homework, or communicating with teachers) [22], which support students’ educational progress. However, the multiple definitions of PI [23] in this study are conceptualized as participation in school-related activities, and communication is both an involvement and a means of improving PI [24].

Joyce Epstein [25], focused on creating and strengthening the bonds between school, family, and community, developing the most widely cited and researched PI model in school. In order to foster the growth of students’ success in school in different domains (for example, school, social, relational and behavioral), Epstein’s [25] model established six types of PI: (i) parenting, which consists of taking care of the health and safety of children, developing good parenting skills in training and preparing children for school and providing a peaceful situation at home, allowing children to focus on their learning activities and complete their studies and making their homework; (ii) communicating, which involves establishing effective ways of communicating from school to home and from home to school about school programs and progress in children’s learning (e.g., sending messages or letters, phone calls, parents visiting the school, sending news by teachers and directors); (iii) volunteering, which requires the involvement of family members, with available skills to support students in their learning process, inside and outside school; (iv) learning at home, with regards to the relationship between teachers and parents to help students to learn better at home; (v) decision-making, encouraging parents to participate in decision-making to increase student academic performance (e.g., parent participation in the Parent–Teacher Association meeting) and (vi) community collaboration, which involves connections, relationships, and activities able to promote school–family–community collaboration in developing student learning. According to this model, parents, school, and community have spheres of influence shared in the students’ learning process.

### 1.1. Relationships between School Climate, Behaviour Problems and Parental Involvement

The wide and diverse SC-centered research identifies SC as an important factor for increasing the results and improving the school environment. A positive SC will allow students to learn through cooperative learning, team cohesion, and mutual trust [7]. Other SC benefits on the prevention of school violence have also been consistently demonstrated, such as promoting healthy relationships, school connectedness, dropout prevention [26], lower levels of absenteeism (e.g., [27]), and less aggression and violence [28].

An association between SC and BP has also been demonstrated [10], as well the link between SC and school violence [9,29]. In line with this, the research has shown that a decline in SC is related to higher BP at school [30], which include telling lies and breaking rules [31] and externalizing behaviors [3], or even in the increase of absenteeism [27]. Research has shown that inadequate characteristics of the physical environment (e.g., inadequate lighting, hiding places, gang-related or hate-related graffiti, inadequate supervision of corridors, and poor maintenance) can create opportunities for conflict and violence and foster tolerance for aggressive behavior [32]. A meta-analytic review developed by Reaves et al. [10] concluded by a significant relationship between students’ perceptions dimensions of SC and BP over time. More specifically, a relationship between the institutional environment, interpersonal relationships and BP was found; the same not occurring in the other dimensions of the SC, i.e., safety, teaching, and learning, as they were not equally considered in the analyses of the many studies. Furthermore, Aldridge, McChesney, and Afari [33] tried to analyze how SC influenced the prevalence of bullying and delinquent behavior, demonstrating the importance dimensions, such as connection to the school, the existence and clarity of the rules, and the support the teacher, have in preventing victimization experiences and delinquent behavior in the school context. In turn, a longitudinal analysis by Dorio et al. [30] focused on middle school students’ perceptions of SC factors that are associated with bullying participant behaviors in the traditional and cyber contexts found that students’ observations of delinquency and illegal behaviors on school grounds were positively associated with engagement in bullying and outsider behaviors. Absenteeism is another significant chronic problem in schools that is also related to SC factors. Then, a large study by Van Eck et al. [27] in the U.S. with 25,776 middle and high school students from 106 schools, using multilevel latent profile analysis, found that students who consider the SC to be more negative are more likely to have higher rates of absenteeism. As the proportion of students who perceive the SC as “moderate” or “negative” increases, the rates of chronic absence also increase. Other studies intend to analyze the link between the SC dimensions and psychological and behavioral adjustment. For example, a study by Way, Reddy, and Rhodes [31] involving 1451 young adolescents found that a decline in all dimensions of the assessed SC (e.g., teacher and peer support, opportunities for students’ autonomy in the classroom, and clarity and consistency of school rules) was associated with declines over time in psychological and behavioral adjustment. The benefits of increasing SC dimensions in the reduction of certain behavioral problems among peers, such as bullying, have also been documented. In this way, it has been shown that students are less likely to be bullied when they feel a sense of belonging to the school they attend to, thus being more confident, and when they are constantly involved in the classroom [34].

Crime Prevention Through Environmental Design (CPTED) has been identified as a promising approach in redesigning schools, making them safer, more welcoming, and comfortable [35]. Based on an architectural philosophy that aims to prevent criminal or anti-social behavior through the climate, CPTED primarily focused on natural surveillance, access control, and territorial reinforcement [35], considering four main mechanisms: space design, space use, circulation and circulation patterns, and territorial characteristics and space deterioration. The benefits of CPTED in reducing the incidence of BP and school violence have been largely demonstrated (e.g., [32,36]). When analyzing the associations between the physical attributes of schools and violence-related behaviors, Vagi et al. [32], found an association between better scores on the CPTED and a lesser probability of students missing school due to safety issues. School-Wide Positive Behavior Support (SWPBS) constitutes another proactive, quite popular approach that tries to improve the academic and behavioral outcomes for students by targeting the school’s organizational and social culture through different strategies: (a) defining clear expectations for all students at the school; (b) disseminate and promote these expectations to all students; (c) encourage the practice of these expectations; (d) positively reinforce the desired behavior; (e) defining clear consequences for problematic behavior; (f) extending expectations to the whole school; and (g) data collection and use for ongoing decision making [37]. A longitudinal study developed by Wienen et al. [38] with teachers from 23 elementary schools that agreed to participate and started SWPBS implementation found a slight increase in the perception of the prosocial behavior and a decrease in the behavior problems with peers, reporting also different effects in children, teachers, and schools. Despite SWPBS being considered a very promising approach, given its applied nature, it is not without limitations, requiring the development of more studies based on a more robust and rigorous methodology [39].

The PI in students’ schooling has been perceived as having multiple and important benefits [22], including in managing BP initiated in school environments. PI has been successfully associated with increased social and emotional health and to a reduced dropout and substance use [21]), as well as better social functioning [40].

The positive relationship between PI and students’ social functioning is based on several empirical evidences. It has been proven that the greater involvement of parents in the education of their children promotes communication with school personnel about the adjustment and school behavior of children [40]. It allows a better understanding of children’s social difficulties at school, addressing and reinforcing positive behaviors at home, and it makes possible to mediate students’ behavioral difficulties and problems, such as situations of violence and indiscipline in school [41]. The study developed by El Nokali, Bachman, and Votruba-Drzal [40] concluded that PI is associated with a decrease in BP and improvements in children’s social skills. Children with highly involved parents showed better social functioning and less BP, something that was attributed to the fact that communication between parents and teachers focused essentially on the management of students’ social and BP. The research conducted by Thompson et al. [42] on teachers’ perceptions about PI found that teachers rating low PI also reported worse student behavioral indicators, recognizing more externalizing behaviors, fewer social skills, more symptoms of attention deficit, and disturbing behaviors in relation to adults and colleagues. A Portuguese study by Caridade, Azevedo, Dinis, Sani, and Nunes [43] conducted with school personnel participants also found a significant association between PI and the perception of students’ general behavior, with 80% of the professionals rating student’s general behavior as bad, also rating PI as poor.

The literature also shows that a positive SC is more likely to increase PI in children’s school activities. As an example, some studies (e.g., [23,44]) found a strong and positive correlation between SC and PI, concluding that the improvements observed in SC, enhance the involvement of parents in the schooling process. When parents are highly involved in the school community, the SC is more vigilant and favorable [7].

### 1.2. Aims of the Study

Overall, the existing international literature indicates that there are important associations between SC, PI, and BP. However, the majority of the international (e.g., [31,32,45]) and Portuguese [46] research relating these variables under study is focused on students, and the number of studies focusing on parents and teachers [47] or school staff (e.g., [12,15]) is scarce. Furthermore, Portuguese studies that have analyzed the mediating effect of PI on the relationship between SC and BP are unknown. Therefore, this area of research is still opened for further investigation, in order to support the need to improve school instructional and relational practices, identified as predictive of positive academic and social performance [41]. In addition, the systematic and continuous collection of data about SC perception is essential to establish data-driven interventions for safer and healthier schools [48]. Therefore, this study addresses a valuable contribution to the Portuguese literature by comprehensively assessing SC, PI, and BP, investigating whether PI mediates the relationships between SC and BP, considering the school personnel perceptions. More specifically, this study intends to: (i) identify the level of SC, PI, and BP; (ii) determine the relationship between PI, total SC, and its dimensions (i.e., environmental–structural and relationships) and BP; and (iii) investigate the mediation effect of PI on the relationship between total SC and its dimensions (i.e., environmental–structural and relationships) and BP. The following hypotheses are investigated:

**Hypothesis 1** **(H1).** *A positive correlation between the total SC and its dimensions (i.e., environmental–structural and relationships) and PI.*

**Hypothesis 2** **(H2).** *A negative correlation between total SC and its dimensions (i.e., environmental–structural and relationships) and BP.*

**Hypothesis 3** **(H3).** *A negative correlation between PI and BP, is expected.*

**Hypothesis 4** **(H4).** *Low levels/scores of total SC and its dimensions (i.e., environmental–structural and relationships) and PI are expected to be predictors of high levels/scores of BP.*

**Hypothesis 5** **(H5).** *PI is expected to mediate the relationship between SC (i.e., environmental–structural and relationships) and BP.*

## 2. Materials and Methods

### 2.1. Participants

The convenience sample integrated 329 school personnel participants, aged between 29 and 66 years (*M* = 50.78, *SD* = 7.56), mainly female (79%, *n* = 260), and with higher education, 73.6% (*n* = 242). A total of 17.6% (*n* = 58) have secondary education and 8.8% (*n* = 29) have basic education. A substantial percentage of the sample (71.4%, *n* = 235) are teachers, with a significant percentage, 42.9%, having more than 20 years and 34.2% under 10 years of service (Cf. Table 1).

Data were collected from three main locations in Portugal, Lisbon (22.8%, *n* = 75), Porto (38.3%, *n* = 126), and Porto adjacent municipalities (38.9%, *n* = 128) (Cf. Table 1). The option to focus these three different school geographic locations was related to the fact that this study follows a research project conducted in the city of Porto (LookCrim Project). In addition, Lisbon and Porto constitute the two main Portuguese cities which have the highest crime rates. 

### 2.2. Variables and Measures

*Sociodemographic Information.* School personnel were asked to provide background and demographic information including age, sex, marital status, education, school personnel function, school geographic location, and years of professional experience.

*School Climate (SC)* [15]. Participants were asked about environmental–structural dimension, through three variables: school surrounding physical environment, organization/quality of school spaces, and school infrastructural conditions considering the number of students. Similarly, the relationships dimension also included three other variables: dynamics of extracurricular activities developed at school, dynamics of extracurricular activities involving the school and other institutions, and school engagement with the community. For the six variables, school personnel were asked to rate perception using a five-point Likert-type scale (ranging from 1—very poor to 5—very good). Participants were then asked to justify the given answer, selected from a predefined checklist. All five-point Likert scales were recoded in a three-point Likert scale, joining the lowest, i.e., responses 1 and 2, and the highest, i.e., responses 4 and 5, values, to create three distinct groups: poor vs. fair vs. good. The Cronbach’s alpha (*α*) was calculated for each dimension to verify the internal reliability of SC, considering that it is more suited to Likert scales [49] as it is the case in this study. In this sense, the original ratings were added to estimate total SC (*α* = 0.75) and their main two dimensions: environmental–structural (*α* = 0.64) and relationships (*α* = 0.77). The factor analysis reveals the adequacy of the sample through the means of the Kaiser–Meyer–Olkin (KMO) measure of sampling adequacy [50,51] (0.73 > 0.5) and through the Bartlett’s test [49] (*χ*2 = 386.03; *p* < 0.001). The factor loads are above 0.30, varying between 0.30 and 0.72, indicating an adequate level of validity of the selected items.

*Students’ Behavior Problems (BP)* [15]. Participants were asked about students’ behavior, considering three variables: absenteeism, disruptive behavior, and incivilities. Concerning absenteeism, school personnel were requested to rate the perception using a five-point Likert-type scale (ranging from 1—very low to 5—very high). In this study, this variable was dichotomized based on occurrence, i.e., yes for ratings 4 and 5 vs. no for all other ratings. Then, a list of seven disruptive behaviors (e.g., widespread disrespect; disrespect for teachers; disrespect for school personnel; disrespect between students; manifestation of aggressive behavior; tobacco/drug abuse; alcohol consumption) were presented to the participants, who were then asked to identify those behaviors that happened in their school, through yes vs. no responses. An index of disruptive behavior (IDB) was created through the sum of yes responses by participants. A similar strategy was applied regarding incivilities. Participants were asked to select yes vs. no responses upon a predefined list of four items, i.e., scatter/throw trash around the school; destroy/damage equipment; disturbing school functioning; use of inappropriate language. An index of incivilities was then constructed, adding the yes responses. Finally, a global index of behavior problems was established summing the positive responses across all assessed variables, i.e., absenteeism, disruptive behavior, and incivilities.

*Parental Involvement (PI)* [15]. School personnel were asked to rate the perception about the PI in the school activities, using a five-point Likert-type scale (ranging from 1—very low to 5—very high), with one item only.

### 2.3. Procedures

Data collection was accomplished during the school years of 2017/2018 and 2018/2019, through an internet-administered survey. Recruitment of participants occurred through several means, including direct contact with each School Principal by email or phone and a request to forward the questionnaire survey link to the entire school personnel.

The questionnaire was developed as part of the LookCrim research project in which this study is included, based on the reference literature in this area. The instrument was subjected to two validation procedures. First, the questionnaire was analyzed and revised by two senior researchers in this field, who made important contributions, both in terms of the content of the items and in terms of the order in which they are presented to the participants. Subsequently, the questionnaire was subjected to a pre-test accompanied by spoken reflection with a sample of 20 potential participants (10 teachers and 10 non-teachers). The results showed that the instrument proved to be adequate in terms of language and understanding of the items, which did not raise difficulties for the participants to respond.

### 2.4. Ethical Approval

All subjects gave their informed consent for inclusion before participating in the study. Respondents were given a narrative preamble explaining the study and informing them of the voluntary nature of the participation, prior to completing the questionnaires items. The study was conducted in accordance with the Declaration of Helsinki, and the protocol was approved by the Ethics Committee of University Fernando Pessoa (UFP) Porto, Portugal, Project “Diagnosis of the school climate”, 20 April 2017, no specific reference assigned, date acting as reference ID. The research project was also submitted and approved by the Portuguese Ministry of Education (Ref. No. 0498800002).

### 2.5. Data Analyses Strategy

The software IBM Statistical Package for Social Sciences (IBM SPSS for Windows, version 26.0, IBM Corp., Armonk, NY, USA) [52] was used to data analysis. Allowing the characterization of the sample and the identification of the SC, PI, and BP levels (objective i), descriptive univariate analyses were employed. A Pearson correlation analysis was performed to explore the relationship between the variables under study, i.e., SC, PI, and BP (objective ii). Aiming to explore a possible mediating effect between SC, PI, and BP (objective iii), hierarchical regression analyses were also carried out. In order to test mediation between variables, the model 4 of SPSS command Process v3.5, was used, a more robust way to deal with mediation, according to recent literature [53].

## 3. Results

### 3.1. Descriptive Analyses (SC, PI and BP)

Table 2 presents data relating SC, BP, and PI in school based on school personnel perception. The mean for total SC was 20.38 (*SD* = 1.74, range = 12–28), which is in a moderate level. Environmental–structural (*M* = 11.14, *SD* = 1.84) is the higher rated highest SC dimensions. Regarding the different dimensions of BP, the mean of disruptive behavior is the highest (*M* = 2.99, *SD* = 1.84), followed by absenteeism (*M* = 2.60, *SD* = 0.97) and incivilities (*M* = 2.51, *SD* = 1.07).

### 3.2. Relationships between SC and BP: The Mediating Effect of PI

The correlations between the main variables of the study are presented in Table 3. Except for the relationship between absenteeism and environmental–structural SC., it can be verified that all variables are statistically correlated with each other.

Using the Stepwise Method, a multilinear regression was produced with all variables, PI, total SC, and its dimensions, i.e., environmental–structural and relationships, as predictors variables, and BP as the dependent variable. No significant model was found. A significant multilinear model with a nearly significant environmental–structural SC (*β* = −0.099; *p* = 0.058) variable included and PI (*β* = −0.344; *p* < 0.001), explaining 13.8% of BP variance, was found. The environmental–structural SC was not a PI predictor, but it was marginally significant.

Table 4 present the results for the simple regression models obtained with individually significant variables included. The individual predictors of BP found were PI (*β* = −0.359; *p* < 0.001), explaining 12.9% of the variance of BP, total SC (*β* = −0.241; *p* < 0.001), explaining 5.9% of the variance of BP, environmental–structural SC (*β* = −0.152; *p* < 0.001), explaining 2.3% of the variance of BP and relationships SC (*β* = −0.251; *p* < 0.001), explaining 6.3% of the variance of BP.

**Figure 1:**
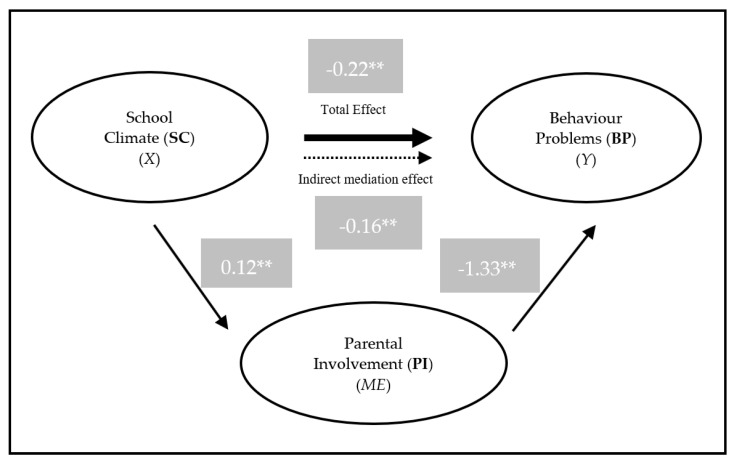
Model of the parental involvement (PI) mediation in the relationship between school climate (SC) and behavior problems (BP). *X*—independent variable; *Y*—dependent variable; *ME*—Mediator; ** *p* < 0.001.

In order to test mediating effects between total SC and its dimensions, i.e., environmental–structural and relationships and BP, the model 4 of SPSS command Process v3.4 [53] (,IBM SPSS for Windows, version 25.0, IBM Corp, Armonk, NY, USA), was used:

The results of PI as mediating the relationship between SC and BP show that it occurs in a full mediating effect. In relation to the effect of SC to BP, there is a significant total effect (*B* = −0.219, *SE* = 0.049, *p* < 0.001), a non-significant direct effect (*B* = −0.0645, *SE* = 0.055, *p* = 0.244), and a significant indirect effect (*B* = −0.155, *p* < 0.001, Bootstrap 95% CI = [−0.2371, −0.1080], BootStrapSE = 0.033), resulting in a full mediation (See Figure 1).

**Figure 2:**
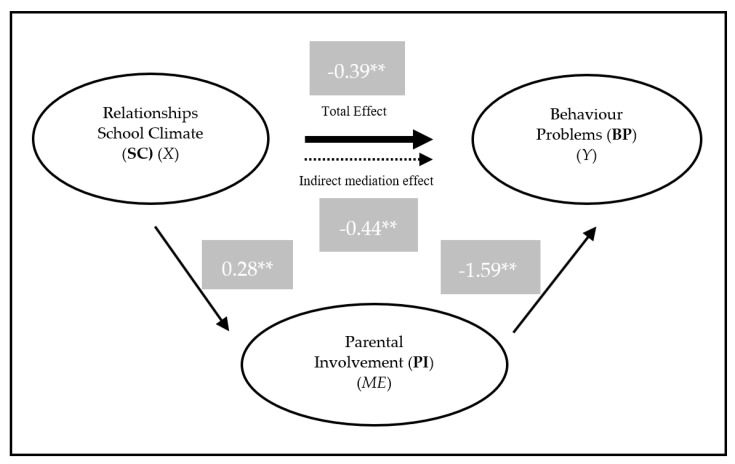
Model of the PI mediation in the relationship between Relationships SC and BP. *X*—independent variable; *Y*—dependent variable; *ME*—Mediator; ** *p* < 0.001.

The results of PI as mediating the relationship between relationships SC and BP show that it occurs in a full mediating effect. In relation to the effect of relationships SC to BP, there is a significant total effect (*B* = −0.385, *SE* = 0.082, *p* < 0.001), a non-significant direct effect (*B* = 0.0521, *SE* = 0.118, *p* = 0.660), and a significant indirect effect (*B* = −0.437, *p* < 0.001, Bootstrap 95% *CI* = [−0.6158, −0.2637], BootStrapSE = 0.090), resulting in a full mediation (See Figure 2).

**Figure 3:**
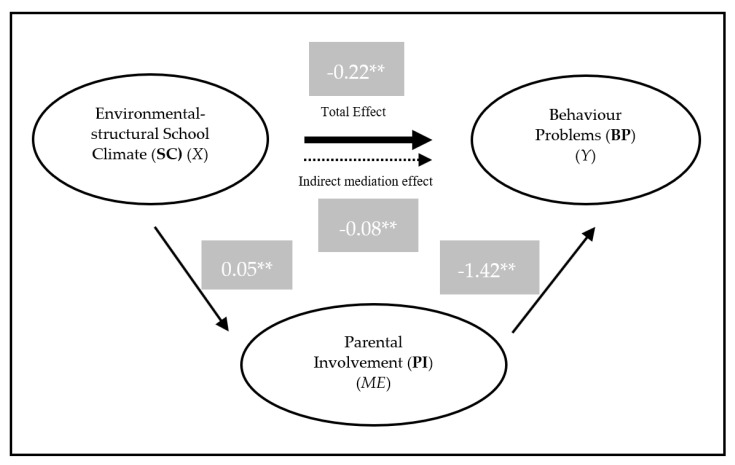
Model of the PI mediation in the relationship between environmental–structural SC and BP. *X*—independent variable; *Y*—dependent variable; *ME*—Mediator; ** *p* < 0.001.

The results of PI as mediating the relationship between environmental–structural SC and BP show that it occurs in a full mediating effect. In relation to the effect of environmental–structural SC to BP, there is a significant total effect (*B* = −0.222, *SE* = 0.080, *p* = 0.006), a non-significant direct effect (*B* = −0.1443, *SE* = 0.076, *p* = 0.058), and a significant indirect effect (*B* = −0.078, *p* < 0.001, Bootstrap 95% *CI* = (−0.1389, −0.0235), BootStrapSE = 0.030), resulting in a full mediation (See Figure 3).

## 4. Discussion

The present study has as main purpose to analyze the mediating effect of PI on the relationships between SC and BP, taking into consideration the school personnel perceptions, a topic that has not been previously studied in the Portuguese context. Similarly, and despite the existence of several international studies focused on these variables proving the existence of a positive relationship SC and BP (e.g., [2,10,29,30,44]) and between SC and PI (e.g., [20,23]), demonstrating that the educational and relational practices of school are important predictors of successful results [42] or even that PI increase social functioning [40] and could yet reduce other problems behaviors (i.e., dropout and substance use) [21], research about mediators is sparce. This study is expected to represent a relevant contribution to the understanding of this phenomenon and to contribute to the development of other studies in this area. Moreover, clarifying the relationship between the variables under study is, therefore, extremely important to implement and improve the level of these variables, SC, PI, and BP, in schools, namely the communication and interaction between family and school and the social functioning of students.

Based on the perception of school personnel, the results of this study allow to conclude by an moderate level of PI and SC, as well as the existence of different BP in the school context (i.e., absenteeism, disruptive behaviors and incivilities), also found in other Portuguese studies [15,16], which could impact in students’ academic achievement [12], as well their social and emotional health [21]. It has been shown that a greater PI in the education of children promotes communication with school personnel [41], allowing for mediation of students’ difficulties and behavioral problems detected in schools, such as situations of violence and indiscipline [41]. In turn, a positive SC has been associated with healthy relationships, a greater school connection, and the prevention of school dropout [26], with the reduction of lower levels of absenteeism [27] and less aggression and violence [28].

It should be highlighted that, with the exception to the relation between absenteeism and environmental–structural SC, all variables under study, i.e., SC, PI, and BP, are statistically correlated with each other. More specifically, PI has a positive important relationship with SC and its dimensions (i.e., environmental–structural and relationships) and a negative significant association with the different BP considered (i.e., disruptive behavior, absenteeism and incivilities). This means that if parents are more involved in school, it is possible to promote SC and decrease BP and, in this way, overall school improvement within the school context performance will be obtained. These results document and reinforce the important role of parents in their children’s school process, whose involvement needs to be widely promoted [54]. In addition, total SC and relationships SC are negatively associated with all variables of BP under study, which means that if the SC is further improved, it will be possible to promote positive changes in student’s behavior [44], also reducing disciplinary and violence problems, such as those demonstrated by other previous evidence through CPTED [32]. In this study, PI emerges as the main predictor of BP, explaining 12.9% of the variance. Total SC and its two dimensions assessed in this study (i.e., environmental–structural and relationships) also emerged as predictive factors for BP, but with low weights (5.9%, 6.3%, and 2.3%, respectively). This result is particularly important in terms of the changes to be implemented in the school context, specifically in terms of the characteristics of physical spaces and the type of relationships promoted at school.

An important finding of this research is related to the mediating effect of PI on the relationship of total SC and its dimensions (i.e., environmental–structural and relationships) and BP, concluding that the SC alone does not explain the improvement of behavior, which is verified only when there is PI. It is clear, therefore, that focusing only on improving school conditions as a strategy to reduce BP in school, may be ineffective in itself. Actions should be combined with promoting PI in their children’s school process more than ever [44].

The present study has some limitations that should be addressed. It is an exploratory study with a cross-sectional nature, focused on school personnel perception comprised by participants which are mainly teachers (70%), and attending only the reports about student’s BP. In future studies, it would be extremely relevant to consider the perceptions of other important actors in the school context, such as parents, students, community members, or even social workers [47] and analyzing records on specific issues about the SC and students’ behavior. Two SC dimensions are being considered in this study. However, because SC is a broader multidimensional construct, future studies should consider other dimensions (e.g., safety, teaching, and learning) to better intervene in the school context and guide reform efforts [11]. The variable PI was only constituted by a generic item, limiting a more detailed exploration of this dimension, something to be addressed in future studies. In the same way, this study only attended to the mediating role of the PI, and future studies should also consider its potential moderating role. Given the importance that the PI has in the students’ school life, future longitudinal studies are necessary in order to better identify the predictors involved. Finally, low alpha values constitute another limitation that should be considered and further explored in future investigations with this questionnaire.

The pandemic problem generated presently by coronavirus COVID-19 that required the implementation of remote learning, highlighted and reinforced the important role of PI in the students’ education, something that should be reflected in further studies analyzing this important particular and extremely relevant period of time.

## 5. Conclusions and Practical Implications

The present study allowed us to highlight the important role that the SC, particularly translated in terms of PI fundamental relevance, has in the decrease of students’ BP, considering the perceptions of the studied school personnel. The focus on teaching professionals (i.e., teachers and non-teachers) constitutes an added value of this paper, since most of the international research has focused mainly on students and, sometimes, on teachers and parents. It was possible to conclude that the influence of the improvement of SC in the management and reduction of BP is particularly visible in the face of the increase in PI. This is an important result that should be consequently considered extremely valuable, influencing the design of policies and strategies to improve the social functioning of students in the Portuguese school context. It is important, therefore, to favor a more ecological approach, focusing on PI, in the context of SC, as a single and combined force to promote interaction between school and family. The lack of time reported by parents has been pointed out as one of the main reasons for reducing PI. The use of social media and online platforms to reach parents and involve them in their children’s school life should be increased. As an example, the use of webinars and discussion forums could be important and empowering tools to achieve this. Encouraging and energizing families to get involved in school activities, as well as carrying out joint activities with children, inside and outside the school context, can improve students’ behavior, with important beneficial implications outside the school context. In addition, the development of support parent networks is another useful strategy for fostering effective participation and decision-making across the school, contributing to enhancing school conditions, as well as providing additional sources of adult monitoring and supervision.

## Figures and Tables

**Table 1:** Participant characteristics (*N* = 329).

Variables	Percentage (*n*)
**Sex**	
Female	79.0 (260)
Male	20.4 (67)
**Education**	
1st–4th year	2.1 (7)
5th–6th year	1.5 (5)
7th–9th year	5.2 (17)
10th–12th year	17.6 (58)
Higher Education	73.6 (242)
**School function**	
Teacher	71.4 (235)
Non-teacher	28.3 (93)
**School geographic location**	
Lisbon	22.8 (75)
Porto	38.3 (126)
Other areas	38.9 (128)

**Table 2:** Descriptive statistics of the variables under study.

Variables/Dimensions	Mean (*SD*)	Total Mean (*M*)	Standard Deviation (*SD*)
**Total School Climate (SC)**		20.38	2.97
Environmental–structural	11.14 (1.84)		
Relationships	9.24 (1.749)		
**Behavior Problems (BP)**		6.05	2.69
Absenteeism	2.60 (0.97)		
Disruptive Behavior	2.99 (1.76)		
Incivilities	2.51 (1.07)		
**Parental Involvement (PI)**	1.68 (0.65)	1.68	0.651

**Table 3:** Pearson’s correlation results for the variables under study (*N* = 329).

Variables	1	2	3	4	5	6	7	8
1. Total School Climate (SC)								
2. Environmental–Structural SC	0.835 **							
3. Relationships SC	0.815 **	0.363 **						
4. Behavior Problems (BP)	−0.242 **	−0.152 **	−0.251 **					
5. Absenteeism	−0.140 *	−0.032	−0.203 **	0.552 **				
6. Disruptive Behavior	−0.199 **	−0.130 **	−0.201 **	0.918 **	0.417 **			
7. Incivilities	−0.216 **	−0.144 **	−0.215 **	0.787 **	0.309 **	0.527 **		
8. Parental Involvement (PI)	0.533 **	0.155 **	0.740 **	−0.359 **	−0.317 **	−0.310 **	−0.276 **	

* *p* < 0.05; ** *p* < 0.001.

**Table 4:** Simple regression analysis with the dependent variable behavior problems.

Predictor Variable Model	*F*(1327)	*R* ^2^	*B*	*β*	*t*
Parental Involvement (PI)	48.387 **	0.129	−1.483	−0.359	−6.956 **
Total School Climate (SC)	20.379 **	0.059	−0.220	−0.242	−4.514 **
Environmental–Structural SC	7.734 *	0.023	−0.222	−0.152	−2.781 **
Relationships SC	21.899 **	0.063	−0.385	−0.251	−4.68 **

Note: *F*: Snedecor’s F-Distribution; *R*^2^: determination coefficient; *B*: unstandardized coefficient; *β*: standardized beta coefficient; *t*: Student’s t-distribution; * *p* < 0.01; ** *p* < 0.001.
